# Testing of symmetric biphasic stimulation in Vim-DBS ET patients: a randomized-controlled pilot study

**DOI:** 10.3389/fneur.2024.1366227

**Published:** 2024-04-24

**Authors:** Alexandra Boogers, Jana Peeters, Tine Van Bogaert, Philippe De Vloo, Wim P. Vandenberghe, Bart Nuttin, Myles Mc Laughlin

**Affiliations:** ^1^Leuven Brain Institute, Leuven, Belgium; ^2^University Hospitals Leuven, Leuven, Belgium

**Keywords:** deep brain stimulation, essential tremor, pulse shapes, biphasic stimulation, cathodic stimulation

## Abstract

**Introduction:**

Symmetric biphasic pulses have been shown to increase the therapeutic window compared to standard cathodic pulses in ET Vim-DBS patients. Furthermore, three hours of stimulation with biphasic pulses caused less stimulation-induced ataxia compared to cathodic pulses. Therefore, an investigation of the longer-term safety of biphasic pulses is warranted.

**Methods:**

Seven ET patients were included in a randomized double-blind, cross-over design of one week home-use of symmetric biphasic stimulation (anodic phase first) versus cathodic stimulation. Amplitude was set in a double-blinded way, at the tremor arrest threshold. The primary outcome was safety assessed by documenting the adverse events. Secondary outcome parameters were stimulation amplitude, tremor (Fahn-Tolosa-Marin Tremor Rating Scale) and ataxia (International Cooperative Ataxia Rating Scale) severity, quality of life (Quality of Life in Essential Tremor Questionnaire) and cognition (Montreal Cognitive Assessment). Three patients continued in the open-label extension phase for 3 months, during which biphasic stimulation-only was further assessed by the same outcome parameters.

**Results:**

During the 1 week testing, no adverse effects were reported. To obtain equivalent tremor control, the amplitude of the biphasic pulse was significantly higher compared to that of the cathodic pulse (*p* = 0.003). The other outcome parameters were not significantly different. During the open-label study, one patient used the remote control to increase the amplitude, leading to two falls caused by stimulation-induced ataxia. No other adverse effects occurred.

**Discussion and conclusion:**

In a small cohort, when tested for one week, symmetric biphasic pulses suggest to be safe, but require higher stimulation amplitudes. Further follow-up studies are needed to investigate long-term effects and safety.

## 1 Introduction

Essential tremor (ET) is the most prevalent movement disorder worldwide (1) and can be severe and pharmaco-refractory, necessitating treatment with deep brain stimulation (DBS) (2). Since the inception of DBS, cathodic pulses have been used (3). In a subset of ET patients, the effect of DBS declines over the course of months to years, a process called ‘habituation’ (4). An increase in stimulation amplitude can render a (sometimes only temporary) tremor suppression with the risk of causing stimulation-induced ataxia (4). Symmetric biphasic pulses have been proposed as a solution to solve the problem of narrow therapeutic windows as these biphasic pulses have shown to mainly increase the side effect threshold compared to cathodic pulses (5). Furthermore, after three hours of symmetric biphasic stimulation, patients had equivalent tremor control but less stimulation-induced ataxia as with cathodic stimulation (6). The mechanisms behind these findings are yet to be determined. Importantly, devices that can stimulate with symmetric biphasic pulses have recently become commercially available (7, 8). To our knowledge, there are, however, no studies describing the long-term safety and effects of biphasic stimulation in clinical DBS. In this study, we investigated the safety and the effect of biphasic stimulation during 1 week, followed by a 3 months open-label follow-up study.

## 2 Methods

Patients with pharmaco-refractory ET, who underwent standard-of-care bilateral Vim-DBS implantation surgery (awake surgery with microelectrode recordings) were recruited. All patients were implanted with Vercise PC and Vercise Gevia internal pulse generators, connected to directional leads (Boston Scientific, Marlborough, MA, United States). The pulses were programmed with study software provided by Boston Scientific. All patients were on stable stimulation parameters for at least three months. All provided written and oral informed consent prior to enrollment. The study was approved by the Ethics Committee of UZ Leuven and conducted in accordance to the Declaration of Helsinki (ClinicalTrials.Gov: NCT04725045).

**Figure 1:**
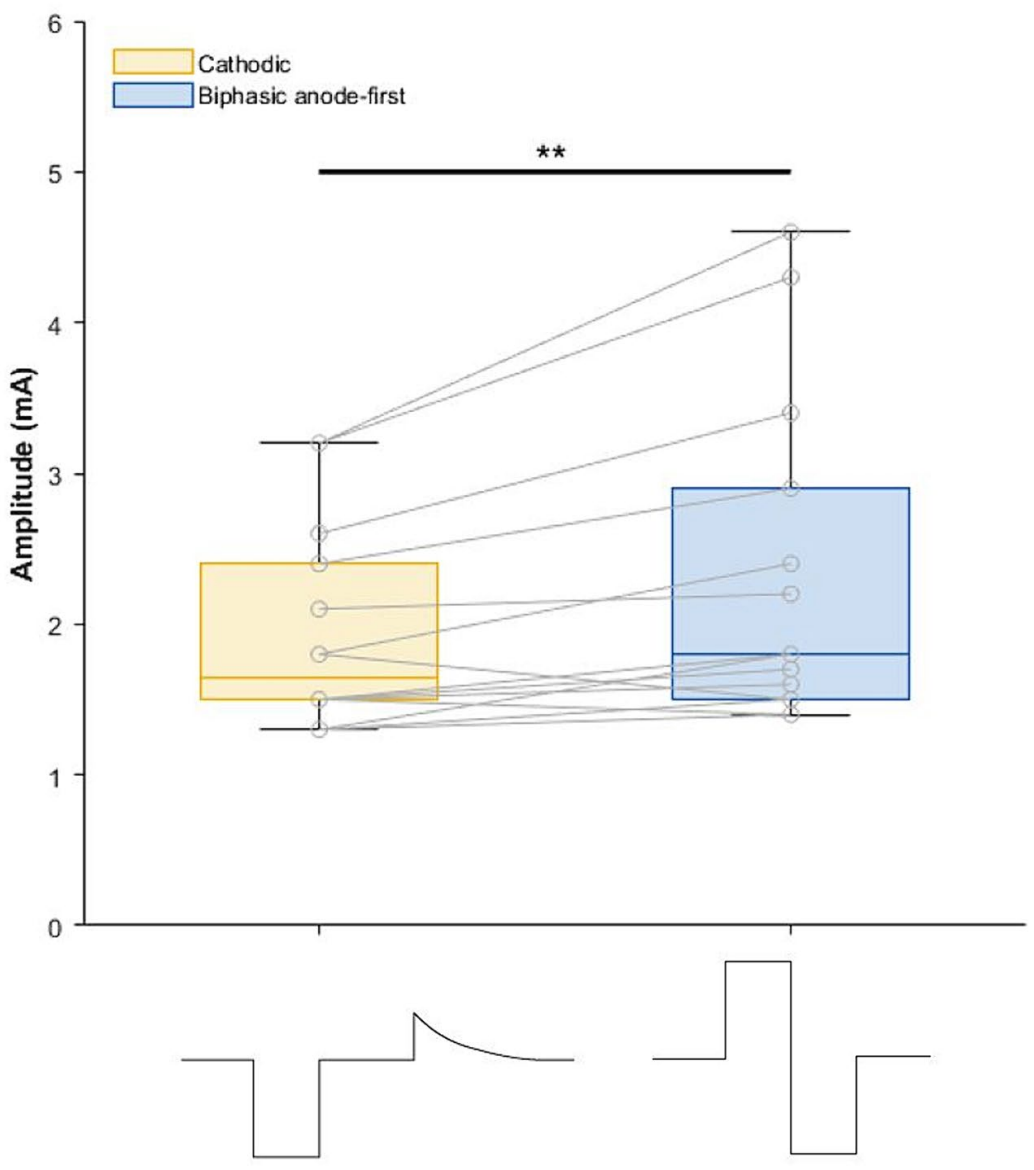
Boxplot depicting amplitude (mA) of cathodic and biphasic pulses during the 1 week home use. On the *x*-axis the pulse shapes are visualized. Recharge phase of cathodic pulse is not drawn to scale. **p = 0.003.

The cathodic pulse, as standardly used in most commercially available devices, consisted of a negatively charged rectangular pulse of 60 μs, followed by a 100 μs interphase gap and a long duration (6 ms), low-amplitude, decaying passive recovery phase. The investigated symmetric biphasic pulse had two rectangular phases of opposite polarity (anodic phase first) of 60 μs each, without an interphase gap (Figure 1). Of note, uptitrating the amplitude of the biphasic pulse was done for both phases simultaneously. The frequency of 130 Hz was used.

### 2.1 Double-blind 1 week cross-over study

Seven patients were included for this part of the study (Supplementary Figure S1). The cross-over study design tested both pulses for 1 week in a random order, without wash-out period in between. The primary outcome was safety. Adverse events were documented during the 2 weeks study period. Secondly, stimulation amplitude was documented by the non-blinded programmer and different clinical outcome measures were collected by the blinded investigator at the end of each one-week stimulation period.

For this phase of the study, the most ventral contact was stimulated bilaterally. If one contact evoked intolerable paresthesia, the second most ventral contact level was selected. The ventral contacts were chosen as they are generally most prone to elicit side effects (9), in the light that the main outcome of this study was safety. Only omnidirectional stimulation was used to limit variability within the study.

At the start of the first week, the lowest clinically effective amplitude of each pulse was determined by the blinded evaluator (blinded for pulse shape and amplitude) by assessing tremor control during a finger-to-nose test, while the non-blinded operator was handling the programming device. The order of definition of the amplitudes of the pulses was random (1:1 randomization via random.org), not necessarily matching the order the one-week stimulation period. Patients were asked not to use their remote control (e.g. for increasing the amplitude) during this part of the study. Compliance was checked at the visit after each week.

The amplitude of the different pulses was compared with a linear mixed-effects model, with subject number and hemisphere number accounted as random effects. Tremor was assessed by the Fahn-Tolosa-Marin Tremor Rating Scale (FTM; sum of part A and part B) and ataxia by the International Cooperative Ataxia Rating Scale (ICARS). Cognition was rated with the Montreal Cognitive Assessment (MoCA). Patients’ quality-of-life was evaluated with the Quality-of-Life in Essential Tremor Questionnaire (QUEST). Daily, at more or less the same moment, the patient was asked to rate the amount of the tremor and the discomfort caused by the tremor on a visual analogue scale (0–10; lower score for better tremor control and less discomfort due to the tremor). Clinical scales were compared between the two pulse shapes with paired Wilcoxon signed rank tests. Statistics were performed in MatLab R2021b (Natick, MA, United States).

### 2.2 Three-month open-label study

After completion of data analysis of the previous study phase, three patients were recruited for the open-label pilot study where safety and efficacy of only the symmetric biphasic pulse was further investigated. Patients were contacted based on distance and accessibility to the hospital. Between these two study phases, patients had been stimulated with their chronic (cathodic) settings, with a wash-out of, respectively, 5, 7, and 10 months. During the open-label study phase, the clinical contact was programmed (directional current steering allowed) as due to the length of the follow-up it was deemed necessary to activate the most effective contact. Patient and evaluator were no longer blinded to pulse shape, nor amplitude. Patients were allowed to change the amplitude with their remote control. Safety was documented by monthly asking for the occurrence of any adverse event. FTM (part A and B), ICARS, MOCA, and QUEST were assessed every month.

## 3 Results

### 3.1 Double-blind cross-over study

**Figure 2:**
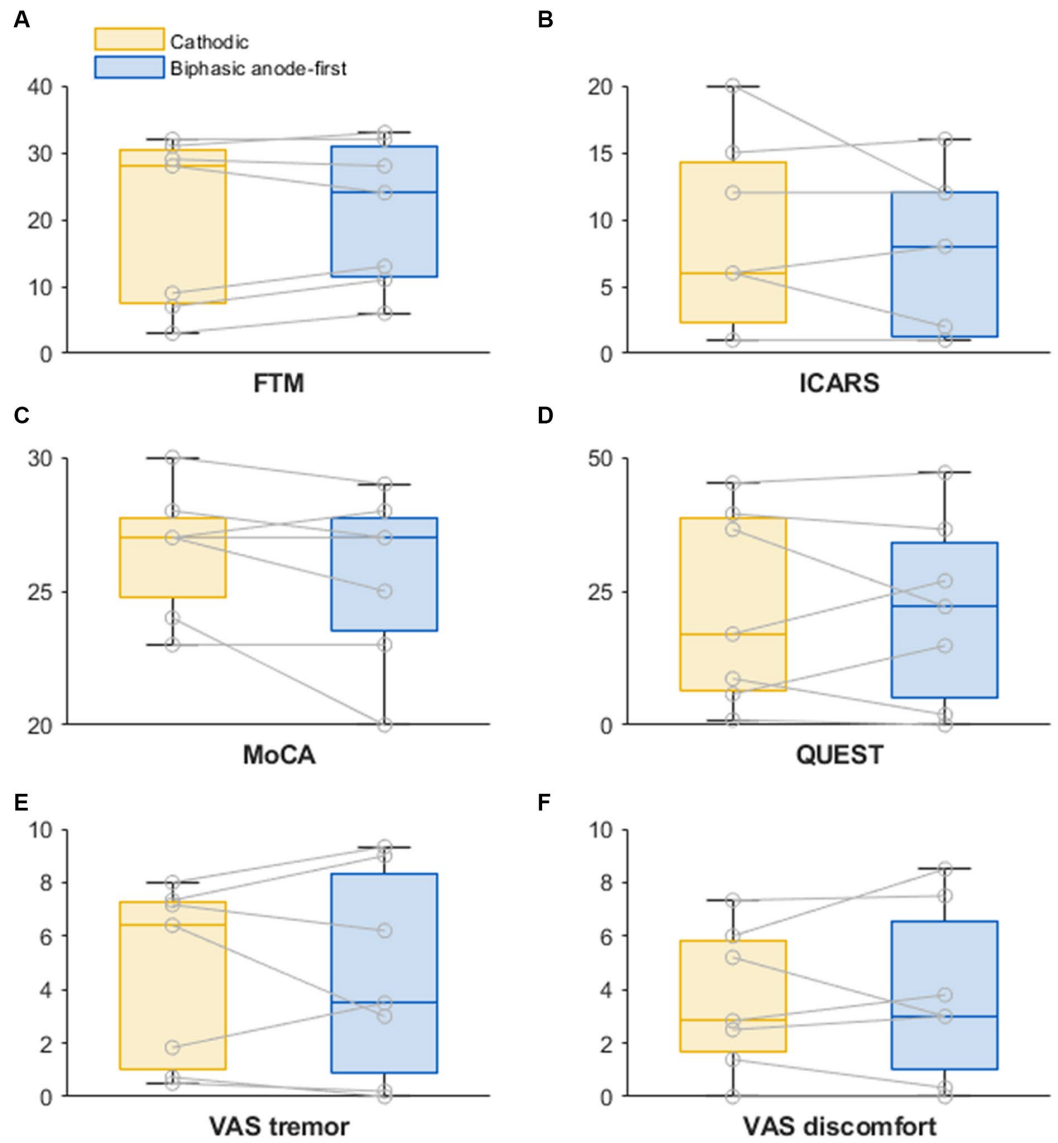
Boxplots of outcomes of **(A)** tremor, **(B)** ataxia, **(C)** cognition, **(D)** quality-of-life, **(E)** VAS rating of tremor, and **(F)** VAS rating of discomfort due to tremor, after 1 week of cathodic and biphasic stimulation (*n* = 7). FTM, Fahn-Tolosa-Marin Tremor Rating Scale; ICARS, International Cooperative Ataxia Rating Scale; MoCA, Montreal Cognitive Assessment; QUEST, quality-of-life in essential tremor questionnaire; VAS, visual analogue scale.

Demographics and stereotactic coordinates of the investigated contacts are described in Table 1. In six hemispheres the most ventral contact was used and in the other eight hemispheres the second most ventral contact was activated. No adverse events were reported during this phase of the study. The stimulation amplitude at tremor arrest amplitude of the biphasic pulse was significantly higher than that of the cathodic pulse 1.93 mA for cathodic threshold (95% confidence interval [1.31; 2.57] and 0.39 mA) threshold increase for the biphasic pulse (95% confidence interval of threshold difference [0.15; 0.64]; *p* = 0.003) (Figure 1). There was no significant difference in tremor or ataxia scores between the cathodic and biphasic pulse (median FTM 28 [range 3–32]) vs. 24 [range 6–33]; *p* = 0.44 and median ICARS 6 [range 1–20] vs. 8 [range 1–16]; *p* = 0.63, respectively. No difference between the pulses was observed in cognition (median MoCA 27 [range 23–30] vs. 27 [range 20–29]; *p* = 0.25) or quality-of-life (median QUEST 17.00 [range 0.96–45.19] vs. 22.12 [range 0–47.12]; *p* = 0.94). Patients’ rating of tremor intensity and discomfort was not different between the two stimulation paradigms either (median VAS for tremor intensity 6.4 [range 0.5–8.0] vs. 3.5 [range 0–9.33]; *p* > 0.99 and median VAS for discomfort 2.83 [range 0–7.3] vs. 3.0 [range 0–8.5]; *p* > 0.99, respectively) (Figure 2; Supplementary Figure S2).

**Table 1:** Demographics of patients included in the study.

Age/gender	Disease duration (years)	Time since surgery (months)	Investigated contact	Coordinates of investigated contact
**One-week follow-up**				
54/M	22	18	Left: ventral Right: ventral	Left: *x*: −12.76 mm; *y*: −6.73 mm; *z*: −0.95 mm Right: *x*: 14.54 mm; *y*: −7.70 mm; *z*: −2.12 mm
65/F	17	12	Left: ventral Right: ventral	Left: *x*: −12.14 mm; *y*: −7.17 mm; *z*: −3.15 mm Right: *x*: 13.23 mm; *y*: −6.30 mm; *z*: −3.79 mm
65/F	45	14	Left: most ventral Right: most ventral	Left: *x*: −13.23 mm; *y*: −6.47 mm; *z*: −1.45 mm Right: *x*: 14.46 mm; *y*: −6.87 mm; *z*: −2.34 mm
81/M	19	8	Left: most ventral Right: most ventral	Left: *x*: −13.30 mm; *y*: −8.02 mm; *z*: 0.66 mm Right: *x*: 10.92 mm; *y*: −9.71 mm; *z*: −5.58 mm
71/M	31	38	Left: most ventral Right: ventral	Left: *x*: −10.17 mm; *y*: −7.83 mm; *z*: −5.46 mm Right: *x*: 12.77 mm; *y*: −7.38 mm; *z*: −2.27 mm
76/M	19	8	Left: most ventral Right: ventral	Left: *x*: −11.29 mm; *y*: −10.26 mm; *z*: −4.75 mm Right: *x*: 12.57 mm; *y*: −10.66 mm; *z*: −2.89 mm
59/M	51	15	Left: ventral Right: ventral	Left: *x*: −13.59 mm; *y*: −4.96 mm; *z*: 1.31 mm Right: *x*: 13.35 mm; *y*: −8.23 mm; *z*: −2.80 mm
**Three-month follow-up**				
71/M	31	50	Left: ventral Right: ventral	Left: *x*: −10.95 mm; *y*: −6.79 mm; *z*: −3.99 mm Right: *x*: 12.77 mm; *y*: −7.38 mm; *z*: −2.27 mm
76/M	19	15	Left: dorsal (directional at 5-c+) Right: most dorsal	Left: *x*: −12.42 mm; *y*: −8.00 mm; *z*: −1.71 mm Right: *x*: 13.96 mm; *y*: −7.91 mm; *z*: −0.43 mm
59/M	51	23	Left: dorsal Right: dorsal	Left: *x*: −14.26 mm; *y*: −3.53 mm; *z*: 2.57 mm Right: *x*: 13.74 mm; *y*: −6.94 mm; z:-1.24 mm

Time since surgery calculated based on timing of start of study phase. Coordinates in native patient space, relative to midcommissural point. Naming of the contacts: most ventral, ventral, dorsal, most dorsal.

### 3.2 Three-month open-label study

Demographics and stereotactic coordinates of the investigated contacts can be found in Table 1. Note that the investigated contacts were located more dorsally in 5/6 hemispheres one level more dorsally (*n* = 3); two levels more dorsally with directional steering (*n* = 1), and three levels more dorsally (*n* = 1). One adverse event was reported: one patient fell twice in the home environment after having increased stimulation amplitude with the remote control due to waning tremor control, resulting in stimulation-induced ataxia. The clinical features of overstimulation could be reversed by decreasing the stimulation amplitude again during the follow-up visit. Otherwise, no adverse events were reported. The amplitude for the biphasic pulse was slightly higher (0.2 to 0.5 mA) compared to the cathodic pulse (Table 2). Clinical outcome parameters of tremor, ataxia, cognition and quality-of-life are described in detail in Table 2.

**Table 2:** Outcome of 3 months open-label extension study.

	Month 0 Cathodic pulse	Month 1 Biphasic pulse	Month 2 Biphasic pulse	Month 3 Biphasic pulse
**Patient 1**				
Amplitude	Vim L: 1.0 mA Vim R: 1.0 mA	Vim L: 1.2 mA Vim R: 1.5 mA	Vim L: 1.5 mA Vim R: 1.8 mA	Vim L: 1.5 mA Vim R: 1.9 mA
FTM	Part A: 5/80 Part B: 7/36	Part A: 5/80 Part B: 11/36	Part A: 3/80 Part B: 8/36	Part A: 4/80 Part B: 8/36
ICARS	3/100	3/100	4/100	3/100
MoCA	30	30	30	30
QUEST	9.62%	7.69%	6.73%	9.62%
AE	–	–	–	–
Adjustments	Month 1: at end of visit, study team increased Vim R by 0.3 mA due to remaining tremor. Month 2: at end of visit, study team increased Vim R by 0.1 mA due to remaining tremor.			
**Patient 2**				
Amplitude	Vim L: 3.2 mA Vim R: 2.1 mA	Vim L: 3.4 mA Vim R: 2.6 mA	Vim L: 3.7 mA Vim R: 2.6 mA	Vim L: 2.9 mA Vim R: 1.8 mA
FTM	Part A: 17/80 Part B: 18/36	Part A: 11/80 Part B: 18/36	Part A: 13/80 Part B: 26/36*	Part A: 12/80 Part B: 13/36
ICARS	10/100	11/100	13/100	9/100
MoCA	28	27	27	28
QUEST	56.73%	53.85%	50.96%	36.54%
AE	–	–	2 non-injurious falls	–
Adjustments	Month 2: patient had increased the amplitude between the visit of month 1 and month 2, leading to ataxic overstimulation. At the end of the study visit of month 2, the study team decreased the stimulation amplitude significantly. *The increase in tremor score was mainly due to worsening of the intentional tremor which was believed to be stimulation-induced (cerebellar overstimulation)			
**Patient 3**				
Amplitude	Vim L: 2.7 mA Vim R: 2.4 mA	Vim L: 2.9 mA Vim R: 2.7 mA	Vim L: 3.1 mA Vim R: 2.9 mA	Vim L: 3.1 mA Vim R: 2.9 mA
FTM	Part A: 7/80 Part B: 20/36	Part A: 11/80 Part B: 21/36	Part A: 4/80 Part B: 17/36	Part A: 5/80 Part B: 17/36
ICARS	8/100	5/100	5/100	6/100
MoCA	22	21	23	24
QUEST	6.73%	20.19%	5.77%	7.69%
AE	–	–	–	–
Adjustments	Month 1: at end of visit, study team increased Vim L by 0.2 mA and R by 0.3 mA due to remaining tremor			

L, left; R, right; FTM, Fahn-Tolosa-Marin Rating Scale; ICARS, International Cooperative Ataxia Rating Scale; MoCA, Montreal Cognitive Assessment; QUEST, quality-of-life in essential tremor questionnaire; AE, adverse event. Note that the reported amplitude correlates to the beginning of the follow-up visit and thus refers to the amplitude at which the assessments were conducted. At the end of the visit, the amplitude could be increased or decreased by the study team to address the patients symptoms better.

## 4 Discussion

### 4.1 Double-blind cross-over study

In this study, the safety of the biphasic pulse during 1 week of stimulation was investigated in seven patients. No adverse events were reported, but we are cautious with our conclusions considering the small sample size. In line with previous results (5, 6), we documented that higher stimulation amplitudes are necessary when stimulating biphasically (symmetric active biphasic pulse with anodic phase first and no interphase gap) to obtain equivalent clinical responses as with cathodic stimulation. This increase in amplitude and the two active phases in symmetric biphasic pulses (active anodic and cathodic phase) theoretically entails an increase of energy consumption compared to the cathodic pulse. This is regarded as the main limitation of biphasic stimulation. Of note, in this study, we did not collect data on battery consumption.

The clinical scales show that the study patients still experienced a moderate amount of tremor after 1 week of stimulation. We propose four possible explanations for this. First, patients were programmed at the lowest clinical benefit which was defined in this study as tremor arrest while performing a finger-to-nose test, which does not necessarily translate into complete tremor control while drawing spirals or pouring water into cups (as is required in part B of the FTM). Secondly, patients were stimulated on their most ventral contact, which is usually not the preferred contact to obtain optimal tremor control (10). Thirdly, in general post-operatively patients experience a reduction in tremor score, though not necessarily a complete tremor arrest. Moreover, tremor control by Vim-DBS decreases over time (11). Lastly, tremor is known to worsen in stressful situations (e.g., a doctor’s visit).

The ataxia scores were not significantly different in this study, contrary to what we published earlier (6). In our previous work, ataxia was intentionally provoked by stimulating at the ataxia threshold of the cathodic pulse. Significantly less ataxia was seen when stimulation at the same amplitude with a biphasic pulse. In the current study, the stimulation amplitudes were much lower, well below the ataxia threshold (defined as ataxic features in finger-to-nose test). Furthermore, the ataxia scores in the present study most likely are influenced by tremor and/or are the consequence of subtle ataxic features seen in people with longstanding essential tremor (9), rather than being the consequence of overstimulation.

There are a few limitations to the study that must be mentioned. Firstly, no wash-out was applied in this cross-over design. However, clinical scales were assessed after one week of stimulation making any carry-over effects unlikely. Secondly, no change in cognition is observed after 1 week of stimulation but due to the short testing interval, a possible (negative) effect on cognition cannot be excluded as a decrease in verbal fluency can be seen in DBS for ET (12), necessitating longer follow-up. Thirdly, the choice for the most ventral or second most ventral contact for this part of the study rather than the clinical contact can be seen as a limitation. The decision to use the ventral contact was guided by safety being the primary outcome of the study. This fourth limitation, together with the stimulation amplitudes at the lowest clinical threshold used in the study, necessitates us to be cautious with the interpretability and generalizability of the study results. It would be interesting to repeat this experiment on the clinically most effective contact as it the current set-up high tremor values are found. Lastly, the sample size is small and hence, our results require confirmation in a larger cohort.

### 4.2 Three-month open-label extension

This pilot study assessed safety and efficacy of 3 months biphasic stimulation on their clinical contact in a small cohort of three patients. The falls of Patient 2 can be attributed to stimulation-induced ataxia caused by the patient increasing the stimulation amplitude with the remote control. This patient was the only person in the cohort known with habituation and stimulation-induced ataxia (including falls) before enrollment. Notably, this event is representative of patients with habituation in whom an increase in stimulation briefly leads to tremor control but is usually followed by increase of intentional tremor (as was documented in this patient, see Table 2) and balance problems due to overstimulation of cerebellar fibers (9). Overstimulation leading to falls is also seen in cathodic stimulation (13), even though we have previously shown that biphasic stimulation can decrease ataxia when compared to cathodic stimulation at the same amplitude (6). The falls could indicate that people with very small therapeutic windows may not safeguarded from overstimulation with biphasic pulses. Therefore, we conclude that symmetric biphasic stimulation need to be tested in a larger cohort and with longer follow-up periods.

## 5 Conclusion

In conclusion, this study is suggestive of safety of the symmetric biphasic pulse (anodic phase first) during 1 week of stimulation. Stimulation with the biphasic pulse required slightly higher amplitudes to obtain equivalent tremor control as with the cathodic pulse. Further research is needed to understand the mechanisms of action of the biphasic pulse and long-term follow-up studies in larger study cohorts are still required.

## Data availability statement

The raw data supporting the conclusions of this article will be made available by the authors, without undue reservation. Requests to access the datasets should be directed to MM, myles.mclaughlin@kuleuven.be.

## Ethics statement

The studies involving humans were approved by UZ Leuven Ethical Board, Belgium. The studies were conducted in accordance with the local legislation and institutional requirements. The participants provided their written informed consent to participate in this study.

## Author contributions

AB: Conceptualization, Formal analysis, Investigation, Methodology, Project administration, Visualization, Writing – original draft, Writing – review & editing. JP: Data curation, Investigation, Writing – review & editing. TB: Data curation, Investigation, Writing – review & editing. PV: Conceptualization, Supervision, Writing – review & editing. WV: Conceptualization, Supervision, Writing – review & editing. BN: Conceptualization, Supervision, Writing – review & editing. MM: Conceptualization, Data curation, Investigation, Methodology, Supervision, Writing – review & editing.
