# Supplementary material for: Testing of symmetric biphasic stimulation in Vim-DBS ET patients: a randomized-controlled pilot study
Source: Front Neurol. 2024 Apr 24;15:1366227. doi: 10.3389/fneur.2024.1366227 (PMC11076781; doi:10.3389/fneur.2024.1366227)
Supplement: Supplementary file 1 [file Image_1.PDF]

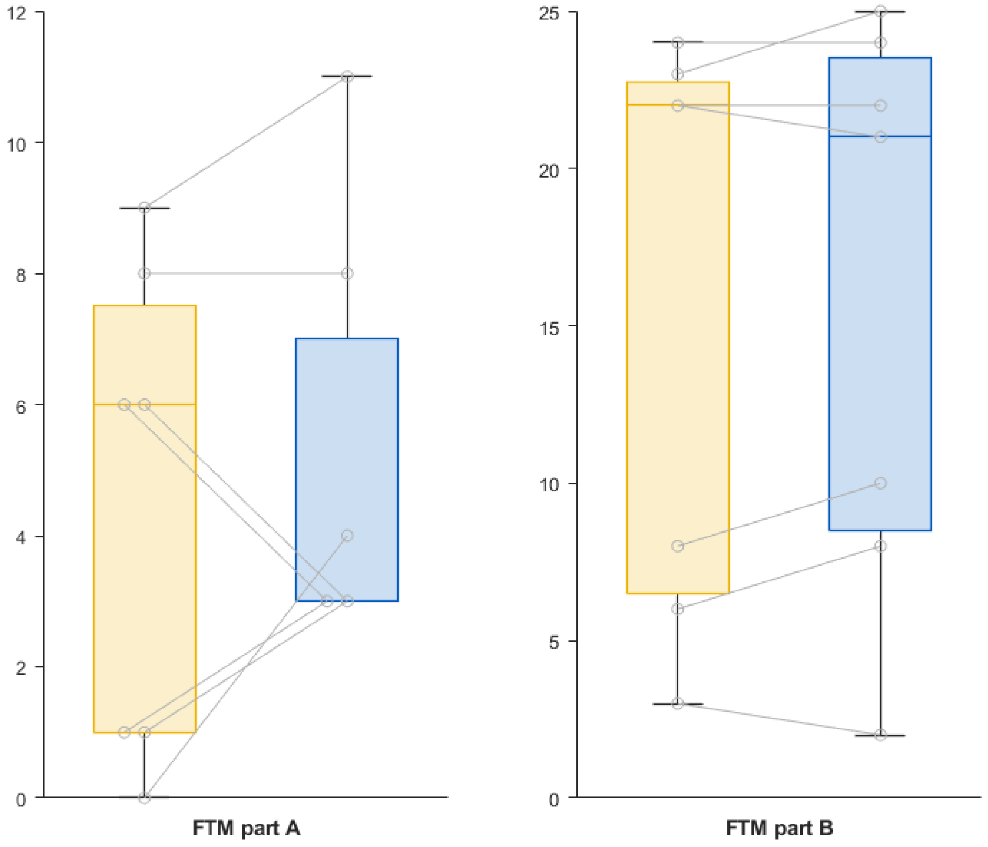

Supplementary Figure 2: Boxplots of the FTM subscores part A and B, after 1 week of cathodic and biphasic stimulation (n = 7). FTM = Fahn-Tolosa-Marin Tremor Rating Scale.
