# Supplementary figures and images for: Testing of symmetric biphasic stimulation in Vim-DBS ET patients: a randomized-controlled pilot study
Source: Front Neurol. 2024 Apr 24;15:1366227. doi: 10.3389/fneur.2024.1366227 (PMC11076781; doi:10.3389/fneur.2024.1366227)

## CONSORT 2010 Flow Diagram

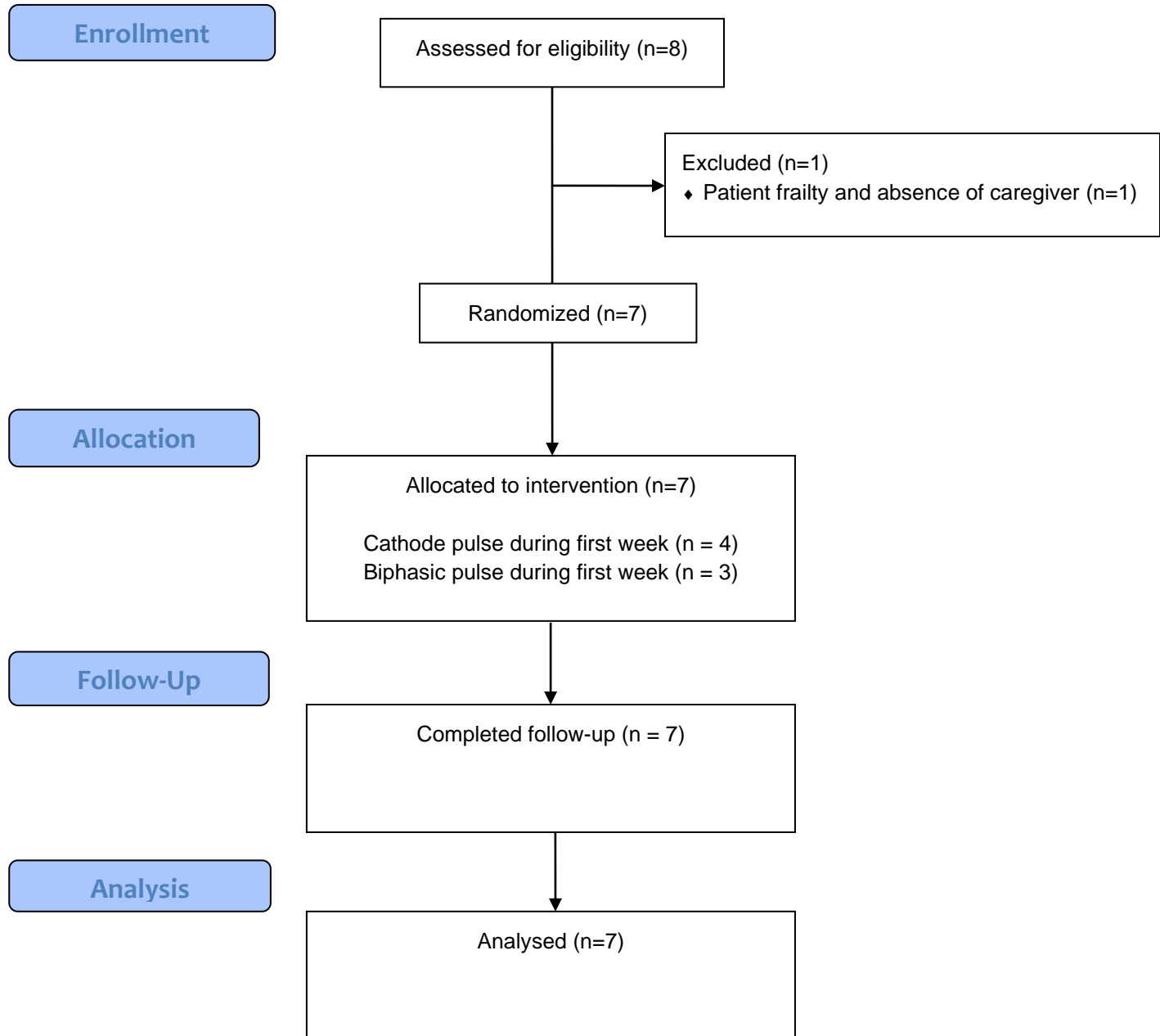

Supplement: Supplementary file 2 [file Data_Sheet_1.PDF]
